# Cross-cultural adaptation of the Parent Hearing Aid Management Inventory into Brazilian Portuguese

**DOI:** 10.1590/2317-1782/20242023249en

**Published:** 2024-09-09

**Authors:** Marília Cardoso Prudêncio, Joseli Soares Brazorotto

**Affiliations:** 1 Department Speech, Language and Hearing Sciences, Associate Postgraduate Program in Speech, Language and Hearing Sciences – PPGFon, Laboratory of Technological Innovation in Health – LAIS, Federal University of Rio Grande do Norte – UFRN - Natal (RN), Brazil.; 2 Postgraduate Program in Health Management and Innovation – PPgGIS, Federal University of Rio Grande do Norte – UFRN - Natal (RN), Brazil.

**Keywords:** Surveys and Questionnaires, Cross-Cultural Comparison, Hearing Aids, Child, Family, Treatment Adherence and Compliance

## Abstract

**Purpose:**

to translate and cross-culturally adapt the Parent Hearing Aid Management Inventory into Brazilian Portuguese.

**Methods:**

study of the methodological type of cross-cultural adaptation, which followed the recommendations of the literature for its execution. Two steps and eight steps were performed to achieve the adaptation: obtaining permission from the authors; formation of a committee of specialists who acted in some of the steps for the validation of the translation, translation by 2 proficient translators, synthesis of the translations and evaluation of equivalences, reverse translation and synthesis of the same, pilot study with 10 families to verify the applicability of the instrument and synthesis of the final version of the instrument.

**Results:**

Cohen's kappa analysis was applied for the inter-rater agreement analysis and Cronbach's alpha coefficient for the analysis of internal reliability of the instrument. After application with the families, the instrument was considered valid to assess the needs for guidance and support of families regarding the management of hearing devices in the population of children with hearing loss.

**Conclusion:**

the Inventory was translated and adapted into Brazilian Portuguese, under the name of Inventário de Manejo dos Aprendidos for the Family (IMAAF) and has the potential to help in clinical practice to achieve effective use of individual sound amplification devices in the population of children with hearing impairment, in a perspective centered on the needs of their families.

## INTRODUCTION

Hearing impairment has a major impact on the life quality of children and their families, as it negatively affects various skills, which can compromise their overall development. Thus, early diagnosis and intervention are essential for better hearing rehabilitation ^(1,2)^, considering that it is through family relationships that children develop ^(3)^, which requires a high level of exposure to essential and quality stimuli ^(4)^.

From the moment of suspicion to the diagnosis of hearing loss, parents experience some phases during this process, feelings that materialize in behaviors and attitudes that directly influence the child's therapeutic process ^(5-7)^. As such, the support network for family members of children with any disability must be solid and well-structured ^(8)^.

Evidence shows that for a good developmental prognosis in this population, conditions such as early identification and diagnosis, an adequate adaptation of hearing devices, their consistent use, specialized therapy, and effective family participation are essential. ^(9,10)^

The literature shows that family members must ensure that the child uses the hearing aid consistently, as well as providing effective hearing and language stimulation ^(11)^. Therefore, the family is the key to treatment adherence.

Consequently, knowing the specific needs of each family in caring for th eir hearing-impaired child is important for organizing a therapeutic plan that considers the needs of each family group, which favors the child's overall development using hearing aids. ^(10,12,13)^

Some families may take a while to understand the dynamics of hearing rehabilitation and consider that just going to weekly therapy sessions is enough, not always encouraging the conscious and effective use of the hearing aid outside the clinical environment. As a result, hopes and expectations of successful development are put to the test ^(14)^. Furthermore, the use of hearing aids can be overestimated by those responsible for them ^(15)^. The potential of each child and their family must therefore be understood, with adjustments to family expectations based on what stimuli they can achieve daily. ^(9, 16)^

Thus, it is the role of the speech therapist to prepare family members, respecting their individuality, cultural diversity, and the way they cope with their child's hearing loss. Counseling is personalized, seeking the independence and autonomy of the family to make assertive decisions about their child's treatment. ^(12,17)^

The application of questionnaires dedicated to analyzing the experience of the family and the child with the devices helps the speech therapist to assess the perceived benefit of using the devices and what are the potential harms that affect the hearing-impaired child and their guardians. In this way, it is possible to offer assertive management of the strategies that involve family dynamics, so that the child achieves the desirable results. In this sense, questionnaires can help professionals in the family counseling process, focusing on their needs, with a greater chance of success in the therapeutic process. ^(10, 13, 18)^

In this sense, the authors Muñoz, *et. al*, 2015 proposed the Parent Hearing Aid Management Inventory (PHAMI), which covers 3 domains that assess the needs and challenges of parents of children with hearing loss in daily situations involving the use of hearing aids.

## PURPOSE

Translate and adapt the Parent Hearing Aid Management Inventory questionnaire into Brazilian Portuguese *- PHAMI* (Muñoz, et. al, 2015).

## METHODS

The current study was approved by the Research Ethics Committee under protocol number 5.924.421.

Parents and/or tutors of hearing-impaired children who used a Hearing Health Center of the Brazilian National Health System (SUS), were selected to engage in this study. All participants signed an Informed Consent Form (ICF).

This is a methodological study of cross-cultural adaptation of an instrument, which was based on the references of validation^(19-23^^)^. In addition, the study followed a set of guidelines for translating and adapting hearing-related questionnaires into different languages^(24)^, thus improving the quality of the translated questionnaire.

### Instrument

The Parent Hearing Aid Management Inventory (PHAMI) developed by Muñoz, *et. al*, 2015 is organized into three sections: Information and Skills (20 questions), Hearing Aid Use (21 questions), and Communication and Support (15 questions). It assesses family members' information and skills in dealing with hearing devices, the frequency of situations that may interfere with the child's use of the hearing device, family members' communication perception, and the support received from the speech therapist on the adaptation and effective use of hearing devices by the child. The focus of the inventory is on Personal Sound Amplification Devices, but it is also applicable to families of children who use other types of electronic hearing aids. Consequently, it is a significant inventory, as it clarifies for the audiologist what guidance, support, and training parents need to be able to support their child's effective use of hearing aids.

The interviewees should check the box for each statement that best describes their need for information and training. The questionnaire doesn't add up to a score, it just helps to detect the vulnerable points for each family, thus aiding the speech therapist's more assertive therapeutic planning.

### Translation, adaptation and cross-cultural validation of the instrument

The process of translation, adaptation, and cross-cultural validation consisted of two stages and eight steps.

#### STAGE 1: Preliminary guidelines

a1) Obtaining permission from the authors of the original test: once the author had agreed, the next stage began;
a2) Creation of a specialist committee (authors of the new version and other specialists) to discuss the concepts surrounding the test to be adapted, considering the group characteristics and culture, made up of two speech therapists with experience in the field of hearing amplification and rehabilitation and a researcher.

#### STAGE 2: Development

b1) Translation: Two qualified translators, native speakers of the target language and fluent in the source language and culture, translated the PHAMI inventory independently, considering conceptual equivalence and avoiding literal translation. Both translators were unfamiliar with the test, one of them not a specialist in the outcome being investigated.
b2) Summary of translations: carried out consensually*, by the same committee mentioned in procedure a2. The committee developed a single version by comparing the translations and assessing semantic, idiomatic, conceptual, linguistic, and contextual discrepancies: semantic equivalence assessed the meaning of words to preserve their original meaning. The idiomatic evaluated the formulation of expressions and colloquialisms equivalent to the target language. Cultural equivalence refers to everyday terms and situations that differ between cultures, and conceptual equivalence refers to words that have different cultural meanings. The judges analyzed the equivalence of the content using a Likert-type scale with scores between -1 and +1, where -1 means not equivalent; 0 means not equivalent; +1 means equivalent. For the item/option to be considered equivalent, it would require to have achieved the four equivalences recommended by the reference adopted in this study, which are: ● Semantic equivalence - equivalence of the grammatical meaning and vocabulary of the words; ● Idiomatic equivalence - use of equivalent expressions in the two languages, English and Portuguese; ^21^ ● Cultural equivalence - coherence with the lived experiences of the population the instrument is intended for; ● Conceptual equivalence - equivalence of the items with the domains they are intended to assess. Whenever the item/option received a score of -1 or 0, the judges had to suggest and justify a change for the item to achieve equivalence. Concerning the analysis, Cohen's kappa was calculated as a quantitative indicator of agreement between the evaluators and Cronbach’s alpha for the reliability of the inventory.
b3) Applicability of the translations/operational equivalence synthesis: the suitability, structure, and application of the items in a real context with 10 families of hearing-impaired children were checked.
b4) Retranslation or reverse translation: the version obtained in procedure b3 was translated into the source language to assess whether the items reflect the content of the original version. The target language version was sent to at least two qualified translators who were unfamiliar with the test, native speakers of the source language, and fluent in the target language and culture.
b5) Synthesis of the retranslated versions: performed consensually*, preferably by the same committee mentioned in procedure a2. The committee compared the retranslations to the original test and evaluated the semantic, idiomatic, conceptual, linguistic, and contextual discrepancies.
b6) Final synthesis: performed consensually*, preferably by the same committee mentioned in procedure a2. The committee compared the original version to the final version in the target language in terms of semantic, idiomatic, conceptual, linguistic, experiential, and contextual equivalence.

## RESULTS

The original version of the Parent Hearing Aid Management Inventory (PHAMI) (Muñoz, *et. al*, 2015) was translated into Brazilian Portuguese and is available in the Appendix A.

Chart 1 shows the original version of the questionnaire, the process of translation, back-translation, and validation by the judges following the suggestions raised, and the result for each item identified for adjustment.

**Chart 1:** Suggestions for adjustments to the PHAMI session items by the expert judges.

**Inventory Sections / Change Suggestions**	**Original Version**	**Portuguese Version**	**Suggestions from expert judges**		**Final version after adjustments**
			**JUDGE 1**	**JUDGE 2**	
**INSTRUCTIONS**	Hearing aids need to be carefully managed for your child to learn how to listen and speak. Your child needs to use well-functioning hearing aids whenever he/she is awake. How your child’s hearing aids are managed every day is a key part of the intervention process and is important for your child’s success. However, managing hearing aids can be hard. You need to learn new information, skills, how to recognize problems, and what to do about problems. The purpose of this questionnaire is to find out what you need to help you manage your child’s hearing aids. This information will help audiologists better meet your needs.	Os aparelhos auditivos precisam ser cuidadosamente gerenciados para que seu filho (a) aprenda a ouvir e falar. Seu filho (a) precisa usar aparelhos auditivos que funcionem bem sempre que ele/ela está acordado. A forma como os aparelhos auditivos do seu filho são manuseados todos os dias é uma parte fundamental do processo de intervenção e é importante para o sucesso do seu filho (a). No entanto, gerenciar aparelhos auditivos pode ser difícil. Você precisa aprender novas informações, habilidades, como reconhecer problemas e o que fazer em relação aos problemas. O objetivo deste questionário é descobrir o que você precisa para ajudá-lo a gerenciar os aparelhos auditivos do seu filho. Essas informações ajudarão os fonoaudiólogos a atender às suas necessidades.	By language structure, sentences​ are shorter; the reading is not so fluid, but the shorter sentences also can facilitate understanding for people with worse​ reading proficiency​.	In the first phrase, I don't know if it's possible because the equivalence of the translation, but is important not to generate expectations, put “so that your son has the opportunity to learn to listen and speak”.	Os aparelhos auditivos precisam ser cuidadosamente gerenciados para que seu filho (a) **possa aprender a ouvir e falar**. Seu filho (a) precisa usar aparelhos auditivos que funcionem bem sempre que ele/ela está acordado. A forma como os aparelhos auditivos do seu filho são manuseados todos os dias é uma parte fundamental do processo de intervenção e é importante para o sucesso do seu filho (a). No entanto, gerenciar aparelhos auditivos pode ser difícil. Você precisa aprender novas informações, habilidades, como reconhecer problemas e o que fazer em relação aos problemas. O objetivo deste questionário é descobrir o que você precisa para ajudá-lo a gerenciar os aparelhos auditivos do seu filho. Essas informações ajudarão os fonoaudiólogos a atender às suas necessidades.
**PART I**					
Item 5	5. Finding options/accessories (e.g., color options, assistive devices, tamper proof battery doors)	5. Como encontrar opções/acessórios diferentes (por exemplo, opções de cores, dispositivos auxiliares, portas de bateria à prova de violação)	5. tamper proof - not a usual vocabulary, perhaps include 'with security try'.	without suggestions	5.Como encontrar opções/acessórios diferentes (por exemplo, opções de cores, dispositivos auxiliares, portas de bateria com trava de segurança.

Item 15	15. Doing a Ling 6 Sound Check (ah, ee, oo, mm, sh, s)	15. Fazer verificação com os sons de *Ling*	15 - Include *Ling* sounds	without suggestions	15. Fazer verificação com os sons de *Ling (/a/, /i/, /u/, /m/, /x/, /s/)*
Item 18	18. Teaching others to help manage the hearing aids (e.g., check function, putting hearing aids on)	18. Saber ensinar outras pessoas a ajudarem a manusear os aparelhos auditivos (por exemplo, professores a verificar a função, colocar aparelhos auditivos.	without suggestions	In item 18, I believe it would be “to teach other people checking​ the devices, for example, check functioning, to put the hearing aids”. The English version is not specifying only the teacher.	18 - **Ensinar outras pessoas** a verificar os aparelhos auditivos (por exemplo, professores a **verificar o funcionamento,** colocar aparelhos auditivos).
Item 20	20. What tools do you have to maintain your child’s hearing aids? (Mark all that apply):	20. Que ferramentas você tem para manter os aparelhos auditivos do seu filho? (Marque todas aquelas que se aplicam):	without suggestions	In item 20, I would put “to carry out maintenance” next to the instead of “keep”. In the tools I would put “to test the battery charge”.	20 - Que ferramentas você tem para **realizar a manutenção** dos aparelhos auditivos do seu filho?
**PART II**					
Itens 16 e 17	16. On good days, my child typically uses his/her hearing aids	16. Em dias bons, meu filho usa o aparelho em média quantas horas/dia	without suggestions	In items 16 and 17, in answer options, the first would be “all the time he/she is awake”.	16. Em dias bons, meu filho usa o aparelho em média quantas horas/ dia?
	( ) All waking hours	( ) Todas as horas			17. Em dias ruins, meu filho usa o aparelho em média quantas horas/ dia?
	17. On difficult days, my child typically uses his/her hearing aids	17. Em dias ruins, meu filho usa o aparelho em média quantas horas/dia			
	( ) All waking hours	( ) Todas as horas			( ) Todas as horas em que ele está acordado
**PART III**			**JUDGE 1**	**JUDGE 2**	
Item 4	4. Checks in with me to see if I need help or support	4. Entra em contato comigo para ver se preciso de ajuda ou suporte	without suggestions	In item 4, I would put “check if I need help” next to instead of “Try to be in contact …".	**4 - Verifica** se preciso de ajuda ou suporte
Item 8	8. Helps me monitor problems until the concern is resolved (e.g., contact is frequent enough to help me tell if I am making progress)	8. Me ajuda a monitorar e entender os problemas até que a preocupação seja resolvida (por exemplo, o contato é frequente o suficiente para me ajudar a saber se estou progredindo)	without suggestions	I found item 8 difficult to understand, perhaps review the way it was written.	8 - Me ajuda a monitorar e entender os problemas até que a preocupação seja resolvida
Additional Comments			JUDGE 1	JUDGE 2	
			without comments	Overall, the questionnaire is equivalent, however, I believe that some writing adjustments can facilitate understanding.	

Two speech therapists specialized in hearing rehabilitation took part in the concordance analysis. A Google Forms® was provided for the judges to analyze semantic equivalence and content validity.

The team of experts who analyzed the translations pointed out that there was equivalence in 46 of the 52 translated items, with 88% consistency. Adjustments were made regarding differences in verbal agreement.

In the introduction, both judges agreed and made specific suggestions for changes, pointing out the equivalence between the original inventory and the translation.

In Part I of the inventory, referring to Instructions and skills, adjustments were suggested by judge 1 in items 5 and 15; and by judge 2 in items 18 and 20, with the other items being considered equivalent.

In Part II of the inventory: Use of the hearing aid, adjustments were suggested to items 16 and 17, while the other items are equivalent.

In Part III of the inventory: on communication and support, the adjustments suggested by the judges were related to items 4 and 8, with the other items being equivalent.

At last, ten family members answered the questionnaire. It was noted that there were difficulties in understanding items 2, 3, and 8 of Part I, requiring an example to facilitate comprehension. There were no difficulties in interpreting the other questions. The families considered the language applied to be adequate.

After answering the questionnaire, the researcher in charge asked the parents about the difficulty and fatigue after the questionnaire. The parents reported that they were already used to answering questionnaires as part of the routine at the hearing rehabilitation service and that they didn't feel any fatigue after applying the inventory in question.

As for the analysis of the instrument's reliability (internal consistency), Cronbach's alpha coefficient was 0.967 for the protocol for the questionnaire answered by the families. The criterion of alpha ≥ 80 was therefore met, indicating almost perfect internal consistency of the reduced version.

Based on the suggestions made by the judges and family members, adjustments were made to the questionnaire and the final version can be found in Appendix B.

Concerning the qualitative descriptive analysis of the answers given by family members, those responsible felt confident about handling, maintaining, and cleaning the device, but when asked about their knowledge concerning the device’s importance for hearing or how it works, they showed that they needed help.

In Part II of the questionnaire, regarding what might interfere with the use of the device, the answer "Never" prevailed in all the answers, demonstrating that the child uses the device, except when asked about the fear of losing or damaging the devices, nine (9) guardians said they were afraid, and this affected the use of the child or adolescent. When asked for other possible reasons for not using the devices, two of them reported a "noisy environment" and "bullying".

In Part III, about contact with the speech therapist, in most questions, those responsible said they were satisfied with the professional's work; however, some would like to see more attempts to contact speech therapists to check on the family members' needs. In addition, there is a growing desire, reported in the questionnaire, for family members to be helped with school and adolescent behavior issues by hearing rehabilitation professionals.

When evaluating the questions about the adherence to the hearing aid, seven (7) family members answered that it was used throughout the day (more than 10 hours), four (4) for most of the day (8-9 hours) and only one (1) answered that it was only used for part of the day (less than 5 hours). When analyzing the data logging of the children whose family members answered the questionnaire, six (6) showed that they used their devices for more than 10 hours a day, two (2) were below the expected 7 hours, the other 3.2 hours and another four children had no recent data on their daily use of the devices, two of whom were cochlear implant users.

## DISCUSSION

Instruments that can measure and score needs and challenges regarding the use and handling of hearing aids by families of hearing-impaired children, especially in Brazil, as proposed in this study, are necessary ^(10-13)^.

In addition, this type of instrument acts as a guide for the speech therapist in creating personalized therapy sessions focused on the challenges reported by family members. ^(9, 12,17)^

Therefore, training and maintaining the skills of those responsible for hearing-impaired children is fundamental to improving their therapeutic management ^(5-8)^.

It is worth noting that the committee of experts and the representatives of the target population were fundamental in guaranteeing the cultural and linguistic equivalence of the inventory for Brazilian Portuguese, helping to reduce cultural and language discrepancies

As limitations of the study, we would highlight the fact that the Portuguese version of the questionnaire was applied to a small number of families, as well as its application in only one region of the country. Studies with its application in all regions would strengthen its cross-cultural adaptation.

Despite these limitations, the Family Hearing Aid Management Inventory was considered by the expert judges to be relevant, feasible, and appropriate.

Participating families understood the questions satisfactorily, with almost perfect internal consistency, which strengthened their validity.

Despite being an inventory aimed at users of Personal Sound Amplification Devices, it can be used with family members of children who use other devices and can be useful in understanding the needs of family members regarding the management and effective use of these devices.

In the original study, the authors proposed that families send their answers to the inventory to the professionals, as a way of being aware of the families' needs beforehand and monitoring those with the greatest difficulties in using the devices effectively. ^(25, 26)^ Since this was a pilot study, during the application of the translated inventory, it was necessary to follow up with the families to evaluate their understanding of the questions. However, the self-applied model proposed in the original study is an interesting possibility for hearing health services to carry out remote monitoring of the pediatric population.

The feasibility of using this tool, which could contribute to managing and improving the use of hearing devices in children with hearing loss, is therefore noteworthy.

## CONCLUSION

The Parent Hearing Aid Management Inventory (PHAMI) has been translated and adapted into Brazilian Portuguese under the name *Inventário de Manejo dos Aparelhos Auditivos pela Família* (IMAAF) (Family Hearing Aid Management Inventory) and has the potential to help clinical practice achieve the effective use of hearing aids in the population of children with hearing loss, from a perspective centered on the needs of their families.

## Appendix A Original version of the Parent Hearing Aid Management Inventory (PHAMI)

HEARING AID MANAGEMENT INVENTORY FOR PARENTS

Hearing aids need to be carefully managed for your child to learn how to listen and speak. Your child needs to use well functioning hearing aids whenever he/she is awake. How your child’s hearing aids are managed every day is a key part of the intervention process and is important for your child’s success. However, managing hearing aids can be hard. You need to learn new information, skills, how to recognize problems, and what to do about problems.

The purpose of this questionnaire is to find out what you need to help you manage your child’s hearing aids. This information will help audiologists better meet your needs.

CHILD'SNAME: _______________________________________________________________ DN: ___/____/______

Device adaptation date:_____/______/________

Relationship with the child. Are you: ( ) Father ( ) Mother ( ) Aunt (o) ( )

How many hours/day do you spend with the child? ____________

Do you participate in any parent support groups? ( ) Yes No

Speech therapist who applied the Inventory:______________Date of application:___/____/____

I. INFORMATION AND SKILLS

Please mark the box for each statement that best describes your needs for information and training.

**Table:**

***Would you like information or help with any of the following:***	**NO** **Got this** **already**	**NO** **Did not get this and do not want it**	**YES** **Got this but I need more help**	**YES** **Did not get this but I want help**
Knowing how to determine if the hearing aids are benefiting my child				
Knowing what my child can and cannot hear without the hearing aids				
Knowing what my child can and cannot hear while wearing hearing aids				
Knowing ways I can prevent losing the hearing aids (e.g., clips to secure aids)				
Finding options/accessories (e.g., color options, assistive devices, tamper proof battery doors)				
Finding financial assistance for costs (e.g., hearing aids, batteries, earmolds, repairs)				
Knowing how to get loaner hearing aids when my child’s hearing aids need to be repaired				
Knowing when the audiologist needs to check the hearing aid settings				
Helping my child hear better in noisy places (e.g., use of an FM system)				
Meeting other parents of children with hearing loss / find parent support organizations				
Knowing how to tell when to change the hearing aid batteries				
Cleaning the earmolds and re-attach the tubing to the hearing aid				
Knowing how to tell when my child needs new earmolds (e.g., earmold is getting loose)				
Using a listening stethoscope to tell when the hearing aid is not working (e.g., weak, distorted)				
Doing a Ling 6 Sound Check (ah, ee, oo, mm, sh, s)				
Doing hearing aid maintenance (e.g., change tone/ear hook, change microphone cover)				
Keeping the hearing aids on when my child resists wearing them				
Teaching others to help manage the hearing aids (e.g., check function, putting hearing aids on)				

What other information and/or skills would help you manage your child’s hearing aids?

____________________________________________________________________________________________________________________________________________________________________________________________________________________________________________________________________________________________________________

What tools do you have to maintain your child’s hearing aids?

( ) Listening stethoscope (attaches to the hearing aid so you can listen to how it sounds)

( ) Battery tester (to check battery function)

( ) Air blower (to blow air through the earmold tubing to remove moisture)

( ) Cleaning tools (to remove wax from the earmold)

II. HEARING AID USE

Please mark the box that best describes how often the following problems interfere with your child using his/her hearing aids.

**Table:**

***How often is your child’s hearing aid use affected by:***	**Never** **a problem**	**Sometimes** **a problem**	**Frequently** **a problem**	**Always** **a problem**
Distractions and needs of other children in the home				
2. Activities (e.g., playing outside, riding in car)				
3. My child not wanting to wear the hearing aids				
4. Difficulty getting a set routine				
5. The hearing aids not working				
6. Other caregivers’ ability to manage the hearing aids				
7. Costs (e.g. batteries, earmolds, repairs)				
8. Concerns about how the hearing aids look				
9. Not seeing my child benefitting from wearing hearing aids				
10. Frequent ear infections				
11. Frequent feedback (whistling/squealing) from the hearing aids				
12. Not being convinced that my child needs to use hearing aids				
13. Pressure from others not to use the hearing aids (e.g., family, other professionals)				
14. Fear of losing or damaging the hearing aids				

15. Please list any other reasons hearing aid use is difficult:

________________________________________________________________________________________________________________________________________________________________________________________________________

16. On good days, my child typically uses his/her hearing aids:

____ all waking hours ____ most of the day (8-9 hours) ____some of the day (5-7 hours) ____ a portion of the day (less than 5 hours)

17. On difficult days, my child typically uses his/her hearing aids:

____ all waking hours ____ most of the day (8-9 hours) ____some of the day (5-7 hours) ____ a portion of the day (less than 5 hours)

18. During the past two weeks, estimate the number that were good days and bad days for hearing aid use: _____ Good days

19. During the past two weeks, estimate the number that were good days and bad days for hearing aid use: _____ Bad days

20. On average how many hours during the day is your child cared for by someone else (e.g., grandparent, day care provider, babysitter)? ______________

III. COMMUNICATION AND SUPPORT

Audiologists can help parents identify problems with hearing aid management and explore solutions. The ways parents want to be supported can vary from person to person. Please mark the box that best describes how well communication with your audiologist is meeting your individual learning and support needs for managing your child’s hearing aids.

**Table:**

When I meet with the audiologist, he/she:	**YES** **My needs are being met**	**YES** **But I want this more often**	**NO** **But I want this**	**NO** **And I don’t want this**
1. Asks for my thoughts and opinions, and listens to what I have to say (e.g., concerns I am having, ideas that I think might help)				
2. Responds to my input in a way that I feel understood (e.g., includes what I have brought up in the discussion/planning)				
3. Is accepting of my challenges (e.g., does not judge me)				
4. Checks in with me to see if I need help or support				
5. Gives me an opportunity to talk about how I am feeling (my emotions)				
6. Helps me recognize what I am doing right				
7. Helps me explore solutions to problems with hearing aid use				
8. Helps me monitor problems until the concern is resolved (e.g., contact is frequent enough to help me tell if I am making progress)				
9. Talks in a way I can understand				
10. Helps me gain confidence in managing my child’s hearing aids (e.g., keeping them on, troubleshooting problems)				
11. Respects my culture and beliefs by taking into account my views				
12. Provides me with concrete resources (e.g., verbally and in writing)				
13. Teaches me in the ways I learn best (e.g., visual, auditory, in writing, hands-on)				

14. What are other ways the audiologist could communicate with you that would help you manage your child’s hearing aids?

____________________________________________________________________________________________________________________________________________________________________________________________________________________________________________________________________________________________________________

## Appendix B Inventário de Manejo dos Aparelhos Auditivos pela Família (IMAAF)

Os aparelhos auditivos precisam ser cuidadosamente gerenciados para que sua criança aprenda a ouvir e falar. Sua criança precisa usar aparelhos auditivos que funcionem bem sempre que está acordada. O manuseio diário dos aparelhos auditivos é uma parte fundamental do processo de intervenção e é importante para o seu sucesso. No entanto, gerenciar aparelhos auditivos pode ser difícil. Você precisa aprender novas informações, habilidades, como reconhecer problemas e o que fazer em relação aos problemas.

O objetivo deste questionário é descobrir o que você precisa para ajudá-lo a gerenciar os aparelhos auditivos de sua criança. Essas informações ajudarão os fonoaudiólogos a atender às suas necessidades.

NOME DA CRIANÇA: _______________________________________________________________ D.N: ___/____/______

Data de adaptação dos dispositivos:______/______/________

Parentesco com a criança. Você é: ( ) Pai ( ) Mãe ( ) Tia (o) ( )

Quantas horas por dia você passa junto à criança? ____________h

Você participa de algum grupo de apoio aos pais? ( ) Sim ( ) Não

Fonoaudiólogo que aplicou o Inventário:________________________________ Data de aplicação:___/____/____

I. INFORMAÇÕES E HABILIDADES

Por favor, marque a caixa para cada afirmação que melhor descreve suas necessidades de informação e treinamento.

**Table:**

	**Não preciso de ajuda, já sei sobre isso**	**Não consigo e não quero ajuda**	**Sim consigo, mas preciso de mais ajuda**	**Não consigo, mas quero ajuda**
1. Saber se os aparelhos estão ajudando minha criança				
2. Saber o que minha criança pode e não pode ouvir **SEM** os aparelhos auditivos				
3. Saber o que minha criança pode e não pode ouvir **COM** os aparelhos auditivos				
4. Conhecer maneiras de evitar a perda dos aparelhos auditivos (por exemplo, clipes para prender os aparelhos)				
5. Como encontrar opções/acessórios diferentes (por exemplo, opções de cores, dispositivos auxiliares, portas de bateria à prova de violação)				
6. Como encontrar assistência financeira para custos (por exemplo, aparelhos auditivos, baterias, moldes, reparos)				
7. Saber como obter aparelhos auditivos emprestados quando os aparelhos auditivos do meu filho (a) precisam ser consertados				
8. Saber quando o fonoaudiólogo precisa verificar as configurações do aparelho auditivo				
9. Ajudar minha criança a ouvir melhor em locais barulhentos (por exemplo, uso de um Sistema de Microfone Remoto)				
10 .Conhecer outros pais de crianças com perda auditiva / encontrar organizações de apoio aos pais				
11. Saber quando trocar as pilhas do aparelho auditivo				
12. Limpar os moldes auriculares e recolocar o tubo no aparelho auditivo				
13. Saber dizer quando minha criança precisa de novos moldes (por exemplo, molde está frouxo)				
14. Saber quando o aparelho auditivo não está funcionando (por exemplo, fraco, distorcido)				
15. Fazer verificação com os sons de *Ling (/a/, /i/, /u/, /m/, /x/, /s/)*				
16. Fazer a manutenção do aparelho auditivo (por exemplo, mudar o gancho da orelha, trocar a tampa do microfone)				
17. Como manter os aparelhos auditivos em minha criança quando ela resiste a usá-los				
18. Saber ensinar outras pessoas a ajudar a manusear os aparelhos auditivos (por exemplo, professores a verificar a função, colocar aparelhos auditivos)				

Que outras informações e/ou habilidades ajudariam você a gerenciar os aparelhos auditivos de sua criança?

____________________________________________________________________________________________________________________________________________________________________________________________________________________________________________________________________________________________________________

Que ferramentas você tem para manter os aparelhos auditivos da sua criança?

( ) Estetoscópio auditivo (se conecta ao aparelho auditivo para que você possa ouvir como soa)

( ) Testador de bateria (para verificar a função da bateria)

( ) Soprador de ar (para soprar ar através do tubo do molde para remover a umidade)

( ) Ferramentas de limpeza (para remover a cera do molde)

II. USO DO APARELHO AUDITIVO

Marque o que melhor descreve a frequência com que os problemas a seguir interferem no uso dos aparelhos auditivos por sua criança.

**Table:**

Com que frequência o uso do aparelho auditivo da sua criança é afetado por:	**NUNCA**	**ÀS VEZES**	**FREQUENTEMENTE**	**SEMPRE**
1. Distrações e necessidades de outras crianças em casa				
2. Atividades (por exemplo, brincar ao ar livre, andar de carro)				
3. Minha criança não quer usar os aparelhos auditivos				
4. Dificuldade em estabelecer uma rotina				
5. Os aparelhos auditivos não funcionam				
6. Capacidade de outros cuidadores (pessoas da família, escola) de gerenciar e manusear os aparelhos auditivos				
7. Custos com aparelho (por exemplo, baterias, moldes, reparos)				
8. Preocupações sobre a aparência dos aparelhos auditivos				
9. Não ver minha criança se beneficiando do uso de aparelhos auditivos				
10. Infecções de ouvido frequentes				
11. Aparelhos apitando frequentemente				
12. Não estar convencido de que minha criança precisa usar aparelhos auditivos				
13. Pressão de outras pessoas para não usar os aparelhos auditivos (por exemplo, família, outros profissionais)				
14. Medo de perder ou danificar os aparelhos auditivos				

Por favor, liste quaisquer outras razões pelas quais o uso efetivo do aparelho auditivo pela criança é difícil.

____________________________________________________________________________________________________

____________________________________________________________________________________________________

____________________________________________________________________________________________________

Em dias bons, minha criança usa o aparelho em média quantas horas/dia?

( ) Todas as horas

( ) Maior parte do dia (8-9 horas)

( ) Um período de tempo (5-7 horas)

( ) Uma parte do dia (menos de 5 horas)

Em dias ruins, minha criança usa o aparelho em média quantas horas/dia?

( ) Todas as horas

( ) Maior parte do dia (8-9 horas)

( ) Um período de tempo (5-7 horas)

( ) Uma parte do dia (menos de 5 horas)

Durante as últimas duas semanas, quantos foram os dias bons para o uso de aparelhos auditivos?

____________________________________________________________________________________________________

Durante as últimas duas semanas, quantos foram os dias ruins para o uso de aparelhos auditivos?

____________________________________________________________________________________________________

Em média, quantas horas durante o dia a criança é cuidada por outra pessoa (por exemplo, avó, babá)?

____________________________________________________________________________________________________

III. COMUNICAÇÃO E SUPORTE

Os fonoaudiólogos podem ajudar os pais a identificar problemas com o gerenciamento de aparelhos auditivos e explorar soluções. A dificuldade encontrada no dia a dia pode variar de pessoa para pessoa. Marque a caixa que melhor descreve a qualidade da comunicação com seu fonoaudiólogo, em que ele atende às suas necessidades individuais de aprendizado e suporte para gerenciar os aparelhos auditivos da sua criança.

**Table:**

Quando eu me encontro com o fonoaudiólogo, ele:	Sim, minhas necessidades são atendidas	Sim, mas deveria ser algo mais frequente	Não, mas eu gostaria que sim	Não e não quero isso
1. Conversa sobre os meus pensamentos e opiniões e ouve o que eu tenho a dizer (por exemplo, preocupações que estou tendo, ideias que acho que podem ajudar)				
2. Responde às minhas sugestões de uma maneira que me sinto compreendida (por exemplo, inclui o que eu trouxe na discussão/planejamento)				
3. Entende meus desafios (não me julga)				
4. Entra em contato comigo para ver se preciso de ajuda ou suporte				
5. Me dá a oportunidade de falar sobre como estou me sentindo (minhas emoções)				
6. Me ajuda a reconhecer o que estou fazendo certo				
7. Me ajuda a explorar soluções para problemas com o uso de aparelhos auditivos				
8. Me ajuda a monitorar e entender os problemas até que a preocupação seja resolvida (por exemplo, o contato é frequente o suficiente para me ajudar a saber se estou progredindo)				
9. Fala de uma maneira que eu consigo entender				
10. Me ajuda a ganhar confiança na gestão dos aparelhos auditivos do meu filho (por exemplo, mantê-los ligados, solucionar problemas)				
11. Respeita minha cultura e crenças, levando em consideração meus pontos de vista				
12. Me fornece recursos concretos (por exemplo, verbalmente e por escrito)				
13. Me ensina da melhor maneira para que eu aprenda (por exemplo, visual, auditivo, por escrito, prático)				

De que outras maneiras o fonoaudiólogo poderia se comunicar com você para ajudá-lo a gerenciar os aparelhos auditivos da sua criança?

____________________________________________________________________________________________________

____________________________________________________________________________________________________________
