# Supplementary material for: Cross-cultural adaptation of the Parent Hearing Aid Management Inventory into Brazilian Portuguese
Source: Codas. 2024 Sep 9;36(4):e20230249. doi: 10.1590/2317-1782/20242023249en (PMC11404983; doi:10.1590/2317-1782/20242023249en)
Supplement: Translation

# Translation: Adaptação transcultural do inventário Parent Hearing Aid Management Inventory para o Português Brasileiro

**DOI:** 10.1590/2317-1782/20242023249pt

**Authors:** Marília Cardoso Prudêncio, Joseli Soares Brazorotto

**Affiliations:** 1 Departamento de Fonoaudiologia, Programa de Pós-Graduação Associado em Fonoaudiologia – PPGFon, Laboratório de Inovação Tecnológica em Saúde – LAIS, Universidade Federal do Rio Grande do Norte – UFRN - Natal (RN), Brasil.; 2 Programa de Pós-Graduação em Gestão e Inovação em Saúde – PPgGIS, Universidade Federal do Rio Grande do Norte – UFRN - Natal (RN), Brasil.

**Keywords:** Inquéritos e Questionários, Comparação Transcultural, Auxiliares de Audição, Criança, Família, Cooperação e Adesão ao Tratamento

## Abstract

**Objetivo:**

traduzir e adaptar transculturalmente o inventário Parent Hearing Aid Management Inventory para o português brasileiro.

**Método:**

estudo do tipo metodológico, de adaptação transcultural, que seguiu as recomendações da literatura para a sua execução. Foram realizadas duas etapas e oito passos para a consecução da adaptação: obtenção de permissão dos autores; formação de um comitê de especialistas que atuaram em alguns dos passos para a validação da tradução, tradução por 2 tradutores proficientes, síntese das traduções e avaliação das equivalências, tradução reversa e síntese das mesmas, estudo piloto com 10 famílias para verificar-se a aplicabilidade do instrumento e síntese da versão final do instrumento.

**Resultados:**

foi aplicada a análise do kappa Cohen para a análise de concordância interavaliadores e o coeficiente de alfa de Cronbach para a análise de confiabilidade interna do instrumento. Após a aplicação com as famílias considerou-se o instrumento válido para avaliar as necessidades de orientação e apoio das famílias quanto ao manejo dos dispositivos auditivos na população de crianças com deficiência auditiva.

**Conclusão:**

o inventário foi traduzido e adaptado para o português brasileiro, sob o nome de Inventário de Manejo dos Aparelhos Auditivos pela Família (IMAAF) e tem o potencial de auxiliar na prática clínica para o alcance do uso efetivo dos aparelhos de amplificação sonora individual na população de crianças com deficiência auditiva, numa perspectiva centrada nas necessidades de suas famílias.

## INTRODUÇÃO

A deficiência auditiva representa um grande impacto na qualidade de vida da criança e de sua família, por afetar negativamente diversas habilidades, o que pode comprometer seu pleno desenvolvimento. Sendo assim, o diagnóstico e intervenção precoces são fundamentais para uma reabilitação auditiva de qualidade ^(1,2)^, considerando que é a partir das relações familiares que a criança se desenvolve ^(3)^, o que exige uma alta exposição a estímulos essenciais e de qualidade ^(4)^.

Do momento da suspeita até o diagnóstico da perda auditiva, os pais vivenciam algumas fases durante este processo, sentimentos que se materializam em comportamentos e atitudes que influenciam diretamente o processo terapêutico da criança ^(5-7)^. Dessa forma, a rede de apoio para os familiares de crianças com qualquer deficiência deve ser sólida e bem estruturada ^(8)^.

Evidências demonstram que para o bom prognóstico de desenvolvimento nesta população, condições como a identificação e diagnóstico precoces, a adaptação adequada dos dispositivos auditivos, seu uso consistente, a terapia especializada e a participação familiar efetiva são essenciais. ^(9,10)^

A literatura evidencia que os familiares devem garantir que a criança utilize o dispositivo auditivo de forma consistente, além de proporcionar uma estimulação efetiva da audição e da linguagem ^(11)^. Portanto, a família é o ponto chave para a adesão ao tratamento.

Deste modo, conhecer as necessidades específicas de cada família para o cuidado com sua criança com deficiência auditiva é importante, para a organização de um planejamento terapêutico que conte com o acolhimento das necessidades de cada grupo familiar, o que favorece o desenvolvimento global da criança usuária de dispositivos auditivos. ^(10,12,13)^

Algumas famílias podem demorar a entender a dinâmica da reabilitação auditiva e consideram que apenas as idas às sessões de terapia semanalmente são suficientes, nem sempre estimulando o uso consciente e efetivo do dispositivo auditivo fora do âmbito clínico. Diante disso, as esperanças e expectativas do sucesso de desenvolvimento são colocadas à prova ^(14)^. Ademais, o uso dos dispositivos auditivos pode ser superestimado por seus responsáveis ^(15)^. Deve-se entender, pois, as potencialidades de cada criança e sua família, com ajustes nas expectativas familiares a partir do que conseguem efetivar de estímulos no dia a dia. ^(9,16)^

Assim, é papel do fonoaudiólogo preparar os familiares, respeitando sua individualidade, diversidade cultural e o modo como enfrentam a deficiência auditiva da criança. O aconselhamento é personalizado, buscando a independência e a autonomia da família para tomar decisões assertivas quanto ao tratamento de seus filhos. ^(12,17)^

Dessa forma, a aplicação de questionários dedicados a analisar a vivência da família e da criança com os dispositivos, auxilia o fonoaudiólogo a avaliar a percepção de benefício do uso dos dispositivos e quais são os potenciais prejuízos que afetam a criança com deficiência auditiva e seus responsáveis. Deste modo, é possível oferecer um gerenciamento assertivo das estratégias que envolvem a dinâmica familiar, para que a criança alcance os resultados desejáveis. Assim, os questionários podem auxiliar os profissionais no processo de orientação familiar, de forma centrada em suas necessidades, com maior chance de sucesso no processo terapêutico. ^(10, 13, 18)^

Neste sentido, os autores Muñoz, et. al, 2015 propuseram o inventário parental *Parent Hearing Aid Management Inventory - PHAMI* (Inventário de Manejo dos Aparelhos Auditivos pela Família), que abrange 3 domínios, os quais avaliam as necessidades e desafios dos pais de crianças com deficiência auditiva nas situações diárias envolvendo o uso dos dispositivos.

## OBJETIVO

Traduzir e adaptar para o português brasileiro o questionário *Parent Hearing Aid Management Inventory - PHAMI* (Muñoz, et. al, 2015).

## MÉTODO

O presente estudo foi aprovado pelo Comitê de Ética Institucional, sob o número de parecer 5.924.421.

Os participantes do estudo são pais e/ou responsáveis de crianças com deficiência auditiva, usuários de um Centro de Saúde Auditiva do Sistema Único de Saúde (SUS). Todos os participantes assinaram o Termo de Consentimento Livre e Esclarecido (TCLE).

Trata-se de pesquisa do tipo metodológica, de adaptação transcultural de instrumento, a qual baseou-se em referenciais de validação de instrumentos^(19-23^^)^ para sua consecução. Ademais, o estudo seguiu um conjunto de diretrizes para tradução e adaptação de questionários relacionados à audição para diferentes idiomas^(24)^, favorecendo uma maior qualidade ao questionário traduzido.

### Instrumento

O inventário parental *Parent Hearing Aid Management Inventory -* PHAMI (Inventário de Manejo dos Aparelhos Auditivos pela Família) desenvolvido por Muñoz, et. al, 2015 é organizado em três seções: Informações e Habilidades (20 questões), Uso do Aparelho Auditivo (21 questões) e Comunicação e Suporte (15 questões). Avalia as informações e habilidades dos familiares para lidar com os dispositivos auditivos, a frequência de situações que podem interferir no uso do dispositivo auditivo pela criança e a percepção dos familiares sobre a comunicação e o suporte recebido do fonoaudiólogo sobre a adaptação e uso efetivo dos dispositivos auditivos pela criança. O foco do inventário é nos Aparelhos de Amplificação Sonora Individual, porém, observa-se que é passível de aplicação em famílias de crianças usuárias de outros tipos de dispositivos eletrônicos auxiliares à audição. Deste modo, é um inventário bastante importante, visto que esclarece ao fonoaudiólogo quais as orientações, apoio e treinamento os pais necessitam para conseguirem sustentar o uso efetivo dos dispositivos auditivos em seu filho (a).

Os respondentes devem assinalar a caixa para cada afirmação que melhor descreve sua necessidade de informação e treinamento. O questionário não soma pontuação, apenas auxilia na detecção dos pontos vulneráveis para cada família, sendo assim, um auxiliar no planejamento terapêutico mais assertivo do fonoaudiólogo.

### Tradução, adaptação e validação transcultural do instrumento

O processo de tradução, adaptação e validação transcultural foi constituído por duas etapas e oito passos.

#### ETAPA 1: Diretrizes prévias

a1) Obtenção de permissão dos autores do teste original: após a anuência do autor, foi iniciada a próxima etapa;
a2) Formação de um comitê de especialistas (autores da nova versão e outros especialistas) para debater os conceitos adjacentes ao teste a ser adaptado, considerando as características da população e cultura-alvo, composto por duas fonoaudiólogas com experiência na área de amplificação e reabilitação auditiva e uma pesquisadora.

#### ETAPA 2: Desenvolvimento

b1) Tradução: Dois tradutores habilitados, nativos no idioma alvo e fluentes no idioma e cultura fonte traduziram o inventário *PHAMI* de forma independente, considerando a equivalência conceitual e evitando a tradução literal. Ambos os tradutores desconheciam o teste, um deles não especialista no desfecho investigado;
b2) Síntese das traduções: feita de forma consensual*, pelo mesmo comitê mencionado no procedimento a2. O comitê construiu uma versão única a partir da comparação das traduções e avaliação das discrepâncias semânticas, idiomáticas, conceituais, linguísticas e contextuais: a equivalência semântica avaliou o significado das palavras na tentativa de preservar seu sentido original. A idiomática, a formulação de expressões e coloquialismo equivalentes ao idioma-alvo. A cultural, os termos e situações cotidianas diferentes entre as culturas e, a equivalência conceitual, referiu-se às palavras que possuíam significados culturais diferentes. A análise da equivalência do conteúdo pelos juízes foi realizada por meio de uma escala do tipo *Likert* que apresenta pontuação entre -1 e +1, em que -1 significa não equivalente; 0 significa pouco equivalente; +1 significa equivalente. Para considerar o item/opção como equivalente ele precisaria ter alcançado as quatro equivalências recomendadas pela referência adotada neste estudo, que são: ● Equivalência semântica - equivalência do significado gramatical e de vocabulário das palavras; ● Equivalência idiomática - uso de expressões equivalentes nos dois idiomas, inglês e português; ^21^ ● Equivalência cultural - coerência com as experiências vivenciadas pela população a que se destina o instrumento; ● Equivalência conceitual - equivalência dos itens com os domínios a que se pretende avaliar. Sempre que o item/opção, recebesse, em seu julgamento, a pontuação -1 ou 0, seria necessário que os juízes fizessem sugestão e justificativa de alteração para que o item alcançasse a equivalência. Para a análise foi realizado o cálculo do *kappa Cohen* como indicador quantitativo de concordância entre os avaliadores e do alfa de *Cronbach* para a confiabilidade do inventário.
b3) Aplicabilidade da síntese das traduções/equivalência operacional: verificou-se a adequação, estrutura e aplicação dos itens em um contexto real, com 10 famílias de crianças com deficiência auditiva;
b4) Retradução ou tradução reversa: a versão obtida no procedimento b3 foi traduzida para o idioma fonte para avaliar se os itens refletem o conteúdo da versão original. A versão no idioma alvo foi encaminhada para pelo menos dois tradutores habilitados que desconheciam o teste, nativos no idioma fonte e fluentes no idioma e cultura alvo;
b5) Síntese das versões retraduzidas: feita de forma consensual*, de preferência pelo mesmo comitê mencionado no procedimento a2. O comitê comparou as retraduções ao teste original e avaliou as discrepâncias semânticas, idiomáticas, conceituais, linguísticas e contextuais;
b6) Síntese final: feita de forma consensual*, de preferência pelo mesmo comitê mencionado no procedimento a2. O comitê comparou a versão original à versão final no idioma alvo quanto às equivalências semântica, idiomática, conceitual, linguística, experiencial e contextual.

## RESULTADOS

A versão original do *Parent Hearing Aid Management Inventory* (*PHAM*I) (Munõz, et. al, 2015) foi traduzida para o português brasileiro e encontra-se disponível no Apêndice A.

O Quadro 1 apresenta a versão original do questionário, o processo de tradução, retrotradução e validação dos juízes com as sugestões apontadas e o resultado para cada item identificado para ajustes.

**Quadro 1:** Sugestões de ajustes nos itens sessões do *PHAMI* pelos juízes especialistas

**Seções do Inventário/Sugestões de Alteração**	**Versão Original**	**Versão Português**	**Sugestões dos Juízes Especialistas**		**Versão Final após ajustes**
			**JUIZ 1**	**JUIZ 2**	
**INSTRUÇÕES**	Hearing aids need to be carefully managed for your child to learn how to listen and speak. Your child needs to use well functioning hearing aids whenever he/she is awake. How your child’s hearing aids are managed every day is a key part of the intervention process and is important for your child’s success. However, managing hearing aids can be hard. You need to learn new information, skills, how to recognize problems, and what to do about problems. The purpose of this questionnaire is to find out what you need to help you manage your child’s hearing aids. This information will help audiologists better meet your needs.	Os aparelhos auditivos precisam ser cuidadosamente gerenciados para que seu filho (a) aprenda a ouvir e falar. Seu filho (a) precisa usar aparelhos auditivos que funcionem bem sempre que ele/ela está acordado. A forma como os aparelhos auditivos do seu filho é manuseada todos os dias é uma parte fundamental do processo de intervenção e é importante para o sucesso do seu filho (a). No entanto, gerenciar aparelhos auditivos pode ser difícil. Você precisa aprender novas informações, habilidades, como reconhecer problemas e o que fazer em relação aos problemas. O objetivo deste questionário é descobrir o que você precisa para ajudá-lo a gerenciar os aparelhos auditivos do seu filho. Essas informações ajudarão os fonoaudiólogos a atenderem às suas necessidades.	Por estrutura da língua, as frases são mais curtas; a leitura não fica tão fluida, mas as frases curtas também podem facilitar a compreensão de pessoas com menor proficiência de leitura	Na primeira frase, não sei se é possível pois a tradução está equivalente, mas para não gerar expectativas irreais, colocar “para que seu filho tenha a possibilidade de aprender a ouvir e falar”	Os aparelhos auditivos precisam ser cuidadosamente gerenciados para que seu filho (a) possa aprender a ouvir e falar. Seu filho (a) precisa usar aparelhos auditivos que funcionem bem sempre que ele/ela está acordado. A forma como os aparelhos auditivos do seu filho é manuseada todos os dias é uma parte fundamental do processo de intervenção e é importante para o sucesso do seu filho (a). No entanto, gerenciar aparelhos auditivos pode ser difícil. Você precisa aprender novas informações, habilidades, como reconhecer problemas e o que fazer em relação aos problemas. O objetivo deste questionário é descobrir o que você precisa para ajudá-lo a gerenciar os aparelhos auditivos do seu filho. Essas informações ajudarão os fonoaudiólogos a atenderem às suas necessidades.
					Os aparelhos auditivos precisam ser cuidadosamente gerenciados para que seu filho (a) **possa aprender a ouvir e falar.**
**PARTE I**			**JUIZ 1**	**JUIZ 2**	
Item 5	5. Finding options/accessories (e.g., color options, assistive devices, tamper proof battery doors)	5.Como encontrar opções/acessórios diferentes (por exemplo, opções de cores, dispositivos auxiliares, portas de bateria à prova de violação)	5 - a prova de violação - não é um vocabulário usual, talvez incluir ‘com try de segurança’.	sem sugestões	5.Como encontrar opções/acessórios diferentes (por exemplo, opções de cores, dispositivos auxiliares, portas de bateria com trava de segurança)
Item 15	15. Doing a Ling 6 Sound Check (ah, ee, oo, mm, sh, s)	15. Fazer verificação com os sons de *Ling*	15 - Incluir sons de *Ling*	sem sugestões	15- **Fazer verificação com os sons de *Ling (/a/, /i/, /u/, /m/, /x/, /s/*** *)*
Item 18	18. Teaching others to help manage the hearing aids (e.g., check function, putting hearing aids on)	18. Saber ensinar outras pessoas a ajudarem a manusear os aparelhos auditivos (por exemplo, professores a verificar a função, colocar aparelhos auditivos)	sem sugestões	No item 18, acredito que seria “ensinar outras pessoas a verificarem os aparelhos auditivos, por exemplo, verificar o funcionamento, colocar os aparelhos auditivos”. No texto em inglês não está especificando somente o professor.	**18 - Ensinar outras pessoas** a verificarem os aparelhos auditivos (por exemplo, professores a **verificar o funcionamento**, colocar aparelhos auditivos)
Item 20	20. What tools do you have to maintain your child’s hearing aids? (Mark all that apply):	20. Que ferramentas você tem para manter os aparelhos auditivos do seu filho?	sem sugestões	No item 20, colocaria “para realizar a manutenção” ao invés de “manter”. Nas ferramentas colocaria “para testar a carga da bateria”.	20 - Que ferramentas você tem para **realizar a manutenção** dos aparelhos auditivos do seu filho?
**PARTE II**			**JUIZ 1**	**JUIZ 2**	
Itens 16 e 17	16. On good days, my child typically uses his/her hearing aids 17. On difficult days, my child typically uses his/her hearing aids ( ) All waking hours	16. Em dias bons, meu filho usa o aparelho em média quantas horas/dia? 17. Em dias ruins, meu filho usa o aparelho em média quantas horas/dia? ( ) todas as horas	sem sugestões	Nos itens 16 e 17, nas opções de resposta, a primeira seria “todo o tempo que está acordado	16. Em dias bons, meu filho usa o aparelho em média quantas horas/dia? 17. Em dias ruins, meu filho usa o aparelho em média quantas horas/dia? ( ) Todas as horas em **que ele está acordado**
**PARTE III**			**JUIZ 1**	**JUIZ 2**	
Item 4	4. Checks in with me to see if I need help or support	4. Entra em contato comigo para ver se preciso de ajuda ou suporte	sem sugestões	No item 4, colocaria “verifica se preciso de ajuda” ao invés de “entrar em contato…”.	4 - Verifica se preciso de ajuda ou suporte
Item 8	8. Helps me monitor problems until the concern is resolved (e.g., contact is frequent enough to help me tell if I am making progress)	8. Me ajuda a monitorar e entender os problemas até que a preocupação seja resolvida (por exemplo, o contato é frequente o suficiente para me ajudar a saber se estou progredindo)	sem sugestões	Achei o item 8 difícil para entender, talvez rever a forma como foi escrito.	8 - Me ajuda a monitorar e entender os problemas até que a preocupação seja resolvida
**Comentários Adicionais**			**JUIZ 1**	**JUIZ 2**	
			sem comentários	No geral, o questionário é equivalente, no entanto, acredito que alguns ajustes na escrita possam facilitar a compreensão.	

Duas fonoaudiólogas especialistas na área de reabilitação auditiva participaram da análise de concordância. Foi disponibilizado um formulário no *Google Forms®* com o intuito dos juízes realizarem a análise da equivalência semântica e de validade de conteúdo.

A equipe de especialistas que analisou as traduções, apontou que houve equivalência em 46 dos 52 itens traduzidos, com concordância de 88%. Foram realizados ajustes nas diferenças de concordância verbal apontadas.

Na introdução, ambos os juízes estiveram de acordo fizeram sugestões pontuais de alteração, apontando equivalência entre o inventário original e a tradução.

Na Parte I do inventário, referente às Instruções e habilidades foram sugeridos ajustes pelo juiz 1 nos itens 5 e 15 e pelo juiz 2 nos itens 18 e 20, sendo os demais itens considerados equivalentes.

Na Parte II do inventário: Uso do aparelho auditivo foram sugeridos ajustes nos itens 16 e 17, já os demais itens estão equivalentes.

Na Parte III do inventário: sobre comunicação e suporte, os ajustes sugeridos pelos juízes foram relacionados aos itens 4 e 8, estando os demais itens equivalentes.

Na última etapa, dez familiares responderam ao questionário. Observou-se que houve dificuldade para a compreensão nos itens 2, 3 e 8 da Parte I, sendo necessário um exemplo para facilitar a compreensão. Quanto às demais questões, não houve dificuldade de interpretação. As famílias consideraram a linguagem do inventário adequada.

Após responderem ao questionário, a pesquisadora responsável questionou os pais sobre a dificuldade e o cansaço após a aplicação. Os responsáveis relataram que já estão habituados a responderem a questionários na rotina do serviço de reabilitação auditiva, não apresentando fadiga após a aplicação do inventário em questão.

Quanto à análise da confiabilidade (consistência interna) do instrumento, obteve-se o coeficiente alfa de *Cronbach* equivalente a 0.967 para o protocolo para o questionário respondido pelas famílias. Dessa forma, foi atingido o critério de alfa ≥ 80, indicando consistência interna quase perfeita da versão traduzida. A partir das sugestões dos juízes e dos familiares, foram realizados ajustes no questionário alcançando a versão final que se encontra no Apêndice B.

Em relação a análise descritiva qualitativa das respostas dadas pelos familiares, os responsáveis se sentiam seguros quanto ao manuseio, manutenção e limpeza do dispositivo, mas quando perguntados sobre o conhecimento adquirido sobre a importância do aparelho para a audição ou funcionamento do dispositivo, eles demonstraram necessitar de ajuda.

Na Parte II do questionário, sobre o que pode interferir no uso do dispositivo, a resposta "Nunca" prevaleceu em todas as respostas, demonstrando que a criança usa o aparelho, exceto, ao serem perguntados sobre o medo de perder ou danificar os aparelhos, nove (9) responsáveis se demonstraram receosos e isso afetava o uso da criança ou adolescente. Ao solicitar outras possíveis razões para o não uso dos dispositivos, duas delas relataram "ambiente ruidoso" e "*bullying*".

Na Parte III, sobre o contato com o fonoaudiólogo, na grande maioria das perguntas, os responsáveis demonstraram estar satisfeitos com o trabalho do profissional; no entanto, alguns gostariam que houvesse uma quantidade maior de tentativas de contato dos fonoaudiólogos para verificar as necessidades dos familiares. Ademais, há um desejo crescente informado no questionário, para que os familiares sejam auxiliados nas questões escolares e de comportamento dos adolescentes pelos profissionais do serviço de reabilitação auditiva.

Ao avaliar as questões referentes à quantidade de uso do dispositivo auditivo, sete (7) familiares responderam que o uso ocorria durante todo o dia (mais do que 10h), quatro (4) maior parte do dia (8-9 horas) e apenas um (1) respondeu que o uso ocorria apenas durante uma parte do dia (menos de 5 horas). Ao analisar o *data logging* das crianças cujos familiares responderam ao questionário, seis (6) apresentaram uso acima de 10 horas por dia, duas (2) estão abaixo do esperado, sendo 7 horas, e 3,2 horas e de outras duas crianças não havia o dado recente de uso diário dos dispositivos, sendo duas delas usuárias de implante coclear.

## DISCUSSÃO

Instrumentos que consigam mensurar e pontuar necessidades e desafios quanto ao uso e manuseio dos dispositivos auditivos pelas famílias de crianças com deficiência auditiva, especialmente na realidade nacional, proposta da referida pesquisa, são necessários ^(10-13)^.

Ademais, esse tipo de instrumento serve como guia para o profissional fonoaudiólogo na criação de sessões de terapia personalizadas com foco nos desafios relatados pelos familiares. ^(9, 12,17)^

Desta forma, a capacitação dos responsáveis por crianças com deficiência auditiva é fundamental para melhorar seu gerenciamento terapêutico ^(5-8)^.

Vale destacar que o comitê de especialistas e os representantes da população alvo foram fundamentais para garantir a equivalência cultural e linguística do inventário para o português brasileiro, auxiliando na redução das discrepâncias culturais e de linguagem.

Como limitações do estudo, destacamos a aplicação em um número ainda reduzido de famílias da versão em português do questionário, assim como a sua aplicação em uma região do país. Estudos com sua aplicação em todas as regiões fortaleceriam a sua adaptação transcultural.

Apesar destas limitações, o Inventário de Manejo dos Aparelhos Auditivos pela Família foi considerado pelos juízes especialistas como relevante, viável e adequado.

A compreensão das questões pelas famílias participantes foi satisfatória, com consistência interna quase perfeita, o que fortalece sua validade.

Observou-se que, apesar de ser um inventário voltado aos usuários de Aparelhos de Amplificação Sonora Individual, sua aplicação com familiares de crianças usuárias de outros dispositivos é passível de uso e pode ser útil na compreensão das necessidades dos familiares referentes ao manejo e uso efetivo destes dispositivos.

No estudo original, os autores propuseram que as famílias encaminhassem as respostas do inventário aos profissionais, como uma maneira de conhecer previamente as necessidades das famílias e de monitorar aquelas com maiores dificuldades para o uso efetivo dos dispositivos.^(20, 21)^ Por ser um estudo piloto, durante a aplicação do inventário traduzido, foi necessário o acompanhamento junto às famílias, para avaliar a compreensão das questões. Porém, o modelo proposto no estudo original, auto aplicado, é uma possibilidade interessante para que serviços de saúde auditiva façam o acompanhamento à distância na população pediátrica.

Destaca-se, pois, a viabilidade de uso do instrumento apresentado, que poderá contribuir para o gerenciamento e melhora do uso de dispositivos auditivos em crianças com deficiência auditiva.

## CONCLUSÃO

O inventário *Parent Hearing Aid Management Inventory - PHAMI* foi traduzido e adaptado para o português brasileiro, sob o nome de Inventário de Manejo dos Aparelhos Auditivos pela Família (IMAAF) e tem o potencial de auxiliar na prática clínica para o alcance do uso efetivo dos aparelhos de amplificação sonora individual na população de crianças com deficiência auditiva, numa perspectiva centrada nas necessidades de suas famílias.

## Apêndice A Versão original do *Parent Hearing Aid Management Inventory* (*PHAMI*)

HEARING AID MANAGEMENT INVENTORY FOR PARENTS

Hearing aids need to be carefully managed for your child to learn how to listen and speak. Your child needs to use well functioning hearing aids whenever he/she is awake. How your child’s hearing aids are managed every day is a key part of the intervention process and is important for your child’s success. However, managing hearing aids can be hard. You need to learn new information, skills, how to recognize problems, and what to do about problems.

The purpose of this questionnaire is to find out what you need to help you manage your child’s hearing aids. This information will help audiologists better meet your needs.

CHILD'SNAME: _______________________________________________________________ DN: ___/____/______

Device adaptation date:_____/______/________

Relationship with the child. Are you: ( ) Father ( ) Mother ( ) Aunt (o) ( )

How many hours/day do you spend with the child? ____________

Do you participate in any parent support groups? ( ) Yes No

Speech therapist who applied the Inventory:______________Date of application:___/____/____

I. INFORMATION AND SKILLS

Please mark the box for each statement that best describes your needs for information and training.

**Table:**

***Would you like information or help with any of the following:***	**NO** **Got this** **already**	**NO** **Did not get this and do not want it**	**YES** **Got this but I need more help**	**YES** **Did not get this but I want help**
1. Knowing how to determine if the hearing aids are benefiting my child				
2. Knowing what my child can and cannot hear without the hearing aids				
3. Knowing what my child can and cannot hear while wearing hearing aids				
4. Knowing ways I can prevent losing the hearing aids (e.g., clips to secure aids)				
5. Finding options/accessories (e.g., color options, assistive devices, tamper proof battery doors)				
6. Finding financial assistance for costs (e.g., hearing aids, batteries, earmolds, repairs)				
7. Knowing how to get loaner hearing aids when my child’s hearing aids need to be repaired				
8. Knowing when the audiologist needs to check the hearing aid settings				
9. Helping my child hear better in noisy places (e.g., use of an FM system)				
10. Meeting other parents of children with hearing loss / find parent support organizations				
11. Knowing how to tell when to change the hearing aid batteries				
12. Cleaning the earmolds and re-attach the tubing to the hearing aid				
13. Knowing how to tell when my child needs new earmolds (e.g., earmold is getting loose)				
14. Using a listening stethoscope to tell when the hearing aid is not working (e.g., weak, distorted)				
15. Doing a Ling 6 Sound Check (ah, ee, oo, mm, sh, s)				
16. Doing hearing aid maintenance (e.g., change tone/ear hook, change microphone cover)				
17. Keeping the hearing aids on when my child resists wearing them				
18. Teaching others to help manage the hearing aids (e.g., check function, putting hearing aids on)				

*What other information and/or skills would help you manage your child’s hearing aids?*

________________________________________________________________________________________________________________________________________________________________________________________________________

*What tools do you have to maintain your child’s hearing aids?*

( ) Listening stethoscope (attaches to the hearing aid so you can listen to how it sounds)

( ) Battery tester (to check battery function)

( ) Air blower (to blow air through the earmold tubing to remove moisture)

( ) Cleaning tools (to remove wax from the earmold)

II. HEARING AID USE

Please mark the box that best describes how often the following problems interfere with your child using his/her hearing aids.

**Table:**

***How often is your child’s hearing aid use affected by:***	**Never** **a problem**	**Sometimes** **a problem**	**Frequently** **a problem**	**Always** **a problem**
1. Distractions and needs of other children in the home				
2. Activities (e.g., playing outside, riding in car)				
3. My child not wanting to wear the hearing aids				
4. Difficulty getting a set routine				
5. The hearing aids not working				
6. Other caregivers’ ability to manage the hearing aids				
7. Costs (e.g. batteries, earmolds, repairs)				
8. Concerns about how the hearing aids look				
9. Not seeing my child benefitting from wearing hearing aids				
10. Frequent ear infections				
11. Frequent feedback (whistling/squealing) from the hearing aids				
12. Not being convinced that my child needs to use hearing aids				
13. Pressure from others not to use the hearing aids (e.g., family, other professionals)				
14. Fear of losing or damaging the hearing aids				

15. Please list any other reasons hearing aid use is difficult:

_______________________________________________________________________________

_______________________________________________________________________________

16. On good days, my child typically uses his/her hearing aids:

____ all waking hours ____ most of the day (8-9 hours) ____some of the day (5-7 hours) ____ a portion of the day (less than 5 hours)

17. On difficult days, my child typically uses his/her hearing aids:

____ all waking hours ____ most of the day (8-9 hours) ____some of the day (5-7 hours) ____ a portion of the day (less than 5 hours)

18. During the past two weeks, estimate the number that were good days and bad days for hearing aid use: _____ Good days

19. During the past two weeks, estimate the number that were good days and bad days for hearing aid use: _____ Bad days

20. On average how many hours during the day is your child cared for by someone else (e.g., grandparent, day care provider, babysitter)? ______________

III. COMMUNICATION AND SUPPORT

Audiologists can help parents identify problems with hearing aid management and explore solutions. The ways parents want to be supported can vary from person to person. Please mark the box that best describes how well communication with your audiologist is meeting your individual learning and support needs for managing your child’s hearing aids.

**Table:**

When I meet with the audiologist, he/she:	**YES** **My needs are being met**	**YES** **But I want this more often**	**NO** **But I want this**	**NO** **And I don’t want this**
1. Asks for my thoughts and opinions, and listens to what I have to say (e.g., concerns I am having, ideas that I think might help)				
2. Responds to my input in a way that I feel understood (e.g., includes what I have brought up in the discussion/planning)				
3. Is accepting of my challenges (e.g., does not judge me)				
4. Checks in with me to see if I need help or support				
5. Gives me an opportunity to talk about how I am feeling (my emotions)				
6. Helps me recognize what I am doing right				
7. Helps me explore solutions to problems with hearing aid use				
8. Helps me monitor problems until the concern is resolved (e.g., contact is frequent enough to help me tell if I am making progress)				
9. Talks in a way I can understand				
10. Helps me gain confidence in managing my child’s hearing aids (e.g., keeping them on, troubleshooting problems)				
11. Respects my culture and beliefs by taking into account my views				
12. Provides me with concrete resources (e.g., verbally and in writing)				
13. Teaches me in the ways I learn best (e.g., visual, auditory, in writing, hands-on)				

14. What are other ways the audiologist could communicate with you that would help you manage your child’s hearing aids?

________________________________________________________________________________________________________________________________________________________________________________________________________

## Apêndice B Inventário de Manejo dos Aparelhos Auditivos pela Família (IMAAF)

Os aparelhos auditivos precisam ser cuidadosamente gerenciados para que sua criança aprenda a ouvir e falar. Sua criança precisa usar aparelhos auditivos que funcionem bem sempre que está acordada. O manuseio diário dos aparelhos auditivos é uma parte fundamental do processo de intervenção e é importante para o seu sucesso. No entanto, gerenciar aparelhos auditivos pode ser difícil. Você precisa aprender novas informações, habilidades, como reconhecer problemas e o que fazer em relação aos problemas.

O objetivo deste questionário é descobrir o que você precisa para ajudá-lo a gerenciar os aparelhos auditivos de sua criança. Essas informações ajudarão os fonoaudiólogos a atender às suas necessidades.

NOME DA CRIANÇA: _______________________________________________________________ D.N: ___/____/______

Data de adaptação dos dispositivos:______/______/________

Parentesco com a criança. Você é: ( ) Pai ( ) Mãe ( ) Tia (o) ( )

Quantas horas por dia você passa junto à criança? ____________h

Você participa de algum grupo de apoio aos pais? ( ) Sim ( ) Não

Fonoaudiólogo que aplicou o Inventário:________________________________ Data de aplicação:___/____/____

I. INFORMAÇÕES E HABILIDADES

Por favor, marque a caixa para cada afirmação que melhor descreve suas necessidades de informação e treinamento.

**Table:**

	**Não preciso de ajuda, já sei sobre isso**	**Não consigo e não quero ajuda**	**Sim consigo, mas preciso de mais ajuda**	**Não consigo, mas quero ajuda**
1. Saber se os aparelhos estão ajudando minha criança				
2. Saber o que minha criança pode e não pode ouvir **SEM** os aparelhos auditivos				
3. Saber o que minha criança pode e não pode ouvir **COM** os aparelhos auditivos				
4. Conhecer maneiras de evitar a perda dos aparelhos auditivos (por exemplo, clipes para prender os aparelhos)				
5. Como encontrar opções/acessórios diferentes (por exemplo, opções de cores, dispositivos auxiliares, portas de bateria à prova de violação)				
6. Como encontrar assistência financeira para custos (por exemplo, aparelhos auditivos, baterias, moldes, reparos)				
7. Saber como obter aparelhos auditivos emprestados quando os aparelhos auditivos do meu filho (a) precisam ser consertados				
8. Saber quando o fonoaudiólogo precisa verificar as configurações do aparelho auditivo				
9. Ajudar minha criança a ouvir melhor em locais barulhentos (por exemplo, uso de um Sistema de Microfone Remoto)				
10. Conhecer outros pais de crianças com perda auditiva / encontrar organizações de apoio aos pais				
11. Saber quando trocar as pilhas do aparelho auditivo				
12. Limpar os moldes auriculares e recolocar o tubo no aparelho auditivo				
13. Saber dizer quando minha criança precisa de novos moldes (por exemplo, molde está frouxo)				
14. Saber quando o aparelho auditivo não está funcionando (por exemplo, fraco, distorcido)				
15. Fazer verificação com os sons de *Ling*				
16. Fazer a manutenção do aparelho auditivo (por exemplo, mudar o gancho da orelha, trocar a tampa do microfone)				
17. Como manter os aparelhos auditivos em minha criança quando ela resiste a usá-los				
18. Saber ensinar outras pessoas a ajudar a manusear os aparelhos auditivos (por exemplo, professores a verificar a função, colocar aparelhos auditivos)				

Que outras informações e/ou habilidades ajudariam você a gerenciar os aparelhos auditivos de sua criança?

________________________________________________________________________________________________________________________________________________________________________________________________________

Que ferramentas você tem para manter os aparelhos auditivos da sua criança?

( ) Estetoscópio auditivo (se conecta ao aparelho auditivo para que você possa ouvir como soa)

( ) Testador de bateria (para verificar a função da bateria)

( ) Soprador de ar (para soprar ar através do tubo do molde para remover a umidade)

( ) Ferramentas de limpeza (para remover a cera do molde)

II. USO DO APARELHO AUDITIVO

Marque o que melhor descreve a frequência com que os problemas a seguir interferem no uso dos aparelhos auditivos por sua criança.

**Table:**

Com que frequência o uso do aparelho auditivo da sua criança é afetado por:	**NUNCA**	**ÀS VEZES**	**FREQUENTEMENTE**	**SEMPRE**
1. Distrações e necessidades de outras crianças em casa				
2. Atividades (por exemplo, brincar ao ar livre, andar de carro)				
3. Minha criança não quer usar os aparelhos auditivos				
4. Dificuldade em estabelecer uma rotina				
5. Os aparelhos auditivos não funcionam				
6. Capacidade de outros cuidadores (pessoas da família, escola) de gerenciar e manusear os aparelhos auditivos				
7. Custos com aparelho (por exemplo, baterias, moldes, reparos)				
8. Preocupações sobre a aparência dos aparelhos auditivos				
9. Não ver minha criança se beneficiando do uso de aparelhos auditivos				
10. Infecções de ouvido frequentes				
11. Aparelhos apitando frequentemente				
12. Não estar convencido de que minha criança precisa usar aparelhos auditivos				
13. Pressão de outras pessoas para não usar os aparelhos auditivos (por exemplo, família, outros profissionais)				
14. Medo de perder ou danificar os aparelhos auditivos				

Por favor, liste quaisquer outras razões pelas quais o uso efetivo do aparelho auditivo pela criança é difícil.

_______________________________________________________________________________________________________________

_______________________________________________________________________________________________________________

_______________________________________________________________________________________________________________

Em dias bons, minha criança usa o aparelho em média quantas horas/dia?

( ) Todas as horas

( ) Maior parte do dia (8-9 horas)

( ) Um período de tempo (5-7 horas)

( ) Uma parte do dia (menos de 5 horas)

Em dias ruins, minha criança usa o aparelho em média quantas horas/dia?

( ) Todas as horas

( ) Maior parte do dia (8-9 horas)

( ) Um período de tempo (5-7 horas)

( ) Uma parte do dia (menos de 5 horas)

Durante as últimas duas semanas, quantos foram os dias bons para o uso de aparelhos auditivos?

____________________________________________________________________________________________________

Durante as últimas duas semanas, quantos foram os dias ruins para o uso de aparelhos auditivos?

____________________________________________________________________________________________________

Em média, quantas horas durante o dia a criança é cuidada por outra pessoa (por exemplo, avó, babá)?

____________________________________________________________________________________________________

III. COMUNICAÇÃO E SUPORTE

Os fonoaudiólogos podem ajudar os pais a identificar problemas com o gerenciamento de aparelhos auditivos e explorar soluções. A dificuldade encontrada no dia a dia pode variar de pessoa para pessoa. Marque a caixa que melhor descreve a qualidade da comunicação com seu fonoaudiólogo, em que ele atende às suas necessidades individuais de aprendizado e suporte para gerenciar os aparelhos auditivos da sua criança.

**Table:**

Quando eu me encontro com o fonoaudiólogo, ele:	Sim, minhas necessidades são atendidas	Sim, mas deveria ser algo mais frequente	Não, mas eu gostaria que sim	Não e não quero isso
1. Conversa sobre os meus pensamentos e opiniões e ouve o que eu tenho a dizer (por exemplo, preocupações que estou tendo, ideias que acho que podem ajudar)				
2. Responde às minhas sugestões de uma maneira que me sinto compreendida (por exemplo, inclui o que eu trouxe na discussão/planejamento)				
3. Entende meus desafios (não me julga)				
4. Entra em contato comigo para ver se preciso de ajuda ou suporte				
5. Me dá a oportunidade de falar sobre como estou me sentindo (minhas emoções)				
6. Me ajuda a reconhecer o que estou fazendo certo				
7. Me ajuda a explorar soluções para problemas com o uso de aparelhos auditivos				
8. Me ajuda a monitorar e entender os problemas até que a preocupação seja resolvida (por exemplo, o contato é frequente o suficiente para me ajudar a saber se estou progredindo)				
9. Fala de uma maneira que eu consigo entender				
10. Me ajuda a ganhar confiança na gestão dos aparelhos auditivos do meu filho (por exemplo, mantê-los ligados, solucionar problemas)				
11. Respeita minha cultura e crenças, levando em consideração meus pontos de vista				
12. Me fornece recursos concretos (por exemplo, verbalmente e por escrito)				
13. Me ensina da melhor maneira para que eu aprenda (por exemplo, visual, auditivo, por escrito, prático)				

De que outras maneiras o fonoaudiólogo poderia se comunicar com você para ajudá-lo a gerenciar os aparelhos auditivos da sua criança?

____________________________________________________________________________________________________

____________________________________________________________________________________________________
